# Electrochemical Sensor for the Determination of Methylthiouracil in Meat Samples

**DOI:** 10.3390/s22228842

**Published:** 2022-11-15

**Authors:** Andrea Marco, Antonio Canals, Emilia Morallón, Miguel Ángel Aguirre

**Affiliations:** 1 Departamento de Química Física e Instituto Universitario de Materiales, Universidad de Alicante, Apartado 99, 03080 Alicante, Spain; 2 Departamento de Química Analítica, Nutrición y Bromatología e Instituto Universitario de Materiales, Universidad de Alicante, Apartado 99, 03080 Alicante, Spain

**Keywords:** electrochemical sensor, Methylthiouracil, food analysis, screen-printed electrodes, meat

## Abstract

Two analytical methods based on miniaturized electrochemical sensors, voltammetric and amperometric sensors, have been developed for the determination of 6-methyl-2-thiouracil (MTU) in meat consumption samples (beef liver and foie). A multivariate approach has been considered to optimize the experimental procedure including extraction and electrochemical detection. Under optimal conditions and at a typical working potential of 1.55 V (vs Ag pseudo-reference electrode), response is linear in the range 0 to 20 µg L^−1^ MTU concentration range. The empirical limit of detection is 0.13 µg L^−1^, lower than the maximum concentration established by legislation. The electrochemical methods have been used to analyze MTU-spiked meat samples, and recovery values varying between 85 and 95% with coefficients of variation <30%. The analytical methods developed with the miniaturized electrochemical sensors can successfully determine the concentration of MTU in real meat samples with high accuracy, being the results obtained similar to those provided by other methods such as UV-Vis spectrophotometry. Finally, the degree of sustainability of the electrochemical sensors-based developed method has been quantified by means of the Analytical Eco-Scale.

## 1. Introduction

The 6-methyl-2-thiouracil (MTU) is one of the most widely used anti-thyroid drugs to treat hyperthyroidism in both humans and animals, as it inhibits the formation of T3 (triiodothyronine) and T4 (thyroxine) hormones in the thyroid [1,2,3,4,5,6]. If this substance is consumed to a healthy person or animal, it causes hypothyroidism and a decrease in basal metabolism of the body [7]. In short, it causes a faster weight gain, therefore it acts as a growth promoter and its use for this purpose is banned in the European Union [8,9,10,11]. 

Sometimes this drug is clandestinely given to animals in order to make it grow faster and the residues may be left in the animal’s meat or by-products (e.g., milk) [7,12]. In addition, MTU can accumulate in consumers and cause intoxications and even carcinogenic effects [6]. For this reason, it is necessary to carry out regular controls to determine the concentration of MTU in meat and animal by-products intended for human consumption. The maximum allowed concentration of MTU in samples for consumption is, according to the EU directive [9,11], 100 µg L^−1^. This concentration would correspond to a supply of 5 g day^−1^ for 30 days, a period and quantity higher than that stipulated for medicinal use [7].

There are different methods for the determination of this analyte in different matrices, but all include separation or derivatization steps [13,14,15,16,17,18]. Techniques used for quantitative and qualitative MTU determination include chromatographic, electrochemical and colorimetric techniques [13,14,15,16,17,18,19]. For instance, thin layer chromatography (TLC) is used to detect the presence or absence of the analyte and both liquid and gas chromatography are used for quantification. To improve the sensitivity and selectivity of the techniques, mass spectrometer is used for the detection step. However, chromatographic techniques require high-cost instrumentation and high consumption of sample and solvents. To solve these problems, capillary electrophoresis coupled to electrochemiluminescent detection (CE-ECL), a technique capable of separating and detecting thiouracils with high efficiency, is also used [18]. Despite the good results that chromatographic techniques can give in all their variants, the method considered as official by Spanish law is a colorimetric method (UV-Vis spectrophotometry) [19]. In this work, the application of electrochemical techniques for the determination of MTU is investigated.

Electrochemical techniques are a great alternative because they could avoid the separation of the interferents [20]. In addition, they are portable, they require a minimum sample volume in the case of miniaturized electrochemical devices [21,22] and they need low-cost instrumentation. 

Recently, several electrochemical sensors have been developed for several applications [23,24,25]. However, screen-printed electrodes (SPE) have some advantages over conventional electrodes, including the flexibility of the printing design, which allows them to be adapted to the required application [26]. In this work, the application of a miniaturized electrochemical sensor for the determination of MTU is investigated [27,28,29,30] using screen-printed carbon electrode and the comparison with the UV-vis spectrophotometric determination has been studied. Then, different real samples in which the extraction of the MTU before the electrochemical determination have been analyzed. The electrochemical determination of MTU in animal feed using carbon fiber microelectrodes has been studied; however, from our acknowledgment this work is the first time that the MTU is determined in meat real samples using electrochemical techniques.

## 2. Materials and Methods

In this work electrochemical sensors were developed using screen-printed carbon electrodes (Thick-film Carbon Single-Electrodes, Micrux, Oviedo, Spain) to determine MTU concentrations in consumer meat samples acquired in Elche, Spain. These devices consist of a working, a counter and a pseudo-reference electrode, the working and the counter electrodes made from carbon and a silver reference electrode. The working electrode has a geometrical area of 7.1 mm^2^. The active surface area (78.5 cm^2^) was calculated measuring the capacitance in the working electrolyte and considering the capacitance of a non-porous carbon material is 10 µF cm^2^ [31]. All the potentials in this work were referred to an Ag/AgCl/Cl^-^ reference electrode. A SP-200 Biologic Potenciostat (Claix, France) was used for electrochemical determination. Prior to the development of the electrochemical sensors, which require a liquid sample to perform the measurement, it was necessary to perform the extraction of the analyte on solid samples, optimizing and validating the method used. 

Methanol from Merck (Darmstadt, Germany) was used to carry out the extraction procedure. Potassium phosphate (≥99%), dipotassium hydrogen phosphate trihydrate (≥99%) from VWR (Radnor, OH, USA) and ultrapure water (18.2 MΩ·cm) by Milipore^®^ Milli-Q**^®^** water (Burlington, VT, USA) were used to prepare the phosphate buffer solution (PBS) employed as electrolyte at the developed electrochemical sensors. To validate the method, it was necessary to prepare MTU standard solutions using 6-methyl-2-thiouracil from Sigma-Aldrich (Darmstadt, Germany). 

A reference MTU determination method was also carried out to compare the obtained results. Methanol (Merck), chloroform (≥99%) from Sigma-Aldrich, buffer solution at pH 7.6 of tris-(hydroxymethyl) aminomethane by ThermoFisher (Dreieich, Germany), 2,6-dichloroquinone-4-chlorimide (100%) from Sigma-Aldrich, 2-propanol by Supelco, (Darmstadt, Germany) and the use of a Jasco V-670 UV-Vis spectrophotometer (Tokyo, Japan) were required for the realization of this method [19].

### 2.1. MTU Determination from Meat Samples

The MTU extraction procedure used is based on the method for the determination of thiouracils in meat [19] after optimization of several experimental factors [32].

The method followed for the extraction of MTU from meat samples is shown in Figure S1. It was consisted of cutting the sample into 1 cm × 1 cm pieces, weighing 20 g and adding 40 mL of methanol (MeOH). Mechanical agitation was maintained for 10 min to achieve a good homogenization of the extract. Once the extract has been obtained, it was centrifuged for 2 min at 1000 rpm. After centrifugation, the supernatant was collected, however, due to the low surface tension of methanol, it was not possible to carry out the direct MTU determination using the electrochemical sensor. In other words, it did not occur the formation of the drop on the screen-printed electrode (SPE). For this reason, it was necessary to change the medium before carrying out the electrochemical detection. Moreover, to improve the sustainability of the method, a change to aqueous medium was selected, being a 0.1 M phosphate buffer solution (PBS) at pH 7.2. After optimizing the ratio between the extraction medium and the detection medium, the optimum ratio between them was 1:1. 

To reduce the time of the analysis, the effect of the scan rate which determines the oxidation current of MTU has been studied. 

The whole analytical procedure (extraction and electrochemical determination) was performed in triplicate to ensure the repeatability of the method. 

### 2.2. Optimization of the Analytical Method

To determine the optimum conditions of both extraction procedure and the electrochemical detection, a multivariate optimization was performed employing a Plackett-Burmann design in order to identify the significant factors [32]. The software NEMRODW (“New Efficient Methodology for Research Optimal Design”) version 2007/2010 (LPRAI, Marseille, France) was used to design the experimental matrices and to evaluate the results obtained. The recovery factor was used as response and was calculated using the following equation:

(1) $$\mathrm{Recovery}\;\mathrm{factor}\;(\%)=\frac{C_{S}-C_{0}}{C_{A}}\times 100$$

where C_0_, C_A_ and C_S_ represent the concentration of the initial sample, the spiked concentration to the sample and the observed concentration of the spiked sample, respectively. 

Using the factors involved in the determination method, a matrix was constructed (Table S1), from which a small number of randomized experiments (Table S2) were performed. In this work, the experiments are randomly performed to nullify the effect of extraneous or nuisance factors. After the screening study, only one significant factor was found and univariate optimization was carried out by monitoring the effect of this factor (i.e., extractant volume) on the recovery factor values. In these studies, a standard solution containing 50 µg L^−1^ of MTU was used. 

### 2.3. MTU Determination by UV-Vis Spectroscopy

To compare the results obtained using the electrochemical sensors developed, the determination of MTU using a reference method such as UV-Vis spectroscopy was employed (official method).

As in the extraction used for the electrochemical method, the samples had to be cut into 1 cm × 1 cm pieces and weigh 20 g. However, in this procedure the volume of extractant that had to be added is 100 mL of MeOH. Mechanical agitation was maintained for 10 min to achieve a good homogenization of the extract. Once the extract has been obtained, it was centrifuged for 5 min at 2000 rpm. After centrifugation, the supernatant was collected. This is the extraction method described by other authors in bibliography [19].

The determination procedure, summarized in Figure S2, was consisted of evaporating the MeOH extract in a rotary evaporator at 65 °C until the volume was reduced to half the initial volume. After this, the residue was collected in at pH 7.6 buffer solution of tris-(hydroxymethyl) aminomethane and 2 mL of a solution of 2,6-dichloroquinone-4-chlorimide (C_6_H_2_Cl_3_NO) was added. Then it was rested for 20 min so that colour develops in the solution. After this time, it was transferred to a funnel of decanting and a liquid-liquid extraction with 10 mL of chloroform was made. The chloroform extract was collected, and the absorbance was measured at 435 nm. 

This procedure was also performed for the standards, whose absorbance spectra are shown in Figure S3a. 

## 3. Results and Discussion

### 3.1. Preliminary Studies

To check the viability of the developed sensors, the electrochemical activity of the analyte was determined. Other authors [27] demonstrated that MTU presents an oxidation process at 1.35 V in MeOH with the presence of TBAP (tetrabutylammonium perchlorate). However, it was necessary to study the potential at which the analyte undergoes this process in the working aqueous solution. 

This study was performed using an external glassy carbon as working electrode and using both the counter electrode (CE) and the reference electrode (RE) of the screen-printed electrode. 

**Figure 1:**
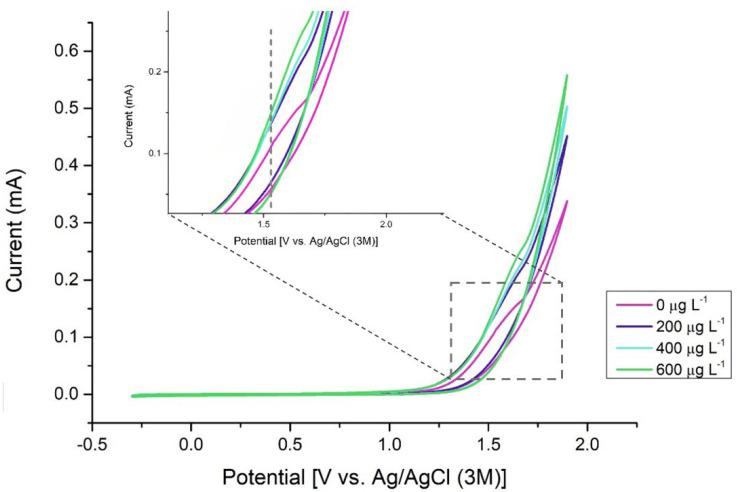
Cyclic voltammogram using a glassy carbon electrode at different MTU concentrations (Scan rate = 50 mV s^−1^). PBS:MeOH 1:1.

By performing cyclic voltammetry of different solutions with increasing concentrations of MTU, the potential at which the oxidation process of the compound occurs was determined. As shown in Figure 1, the current of the oxidation peak, that starts at 1.55 V increases with increasing MTU concentration. 

The 1.55 V potential determined using the external electrode arrangement is the one used to perform the electrochemical determinations by CV and CA on the SPE. The electrochemical characterization is shown in Figures S4 and S5 in the Supplementary Information of this article. In addition, the voltammogram of a real sample is given in Figure S6. The oxidation peaks of uric acid (UA) and ascorbic acid (AA), the two probable interferents, do not appear in this voltammogram.

The electrochemical stability window (ESW) of the PBS:MeOH solution used as electrolyte was determined by cyclic voltammetry in the SPE, being the stable potential window from −0.3 V to 1.9 V.

### 3.2. Optimization of the Analytical Method

**Figure 2:**
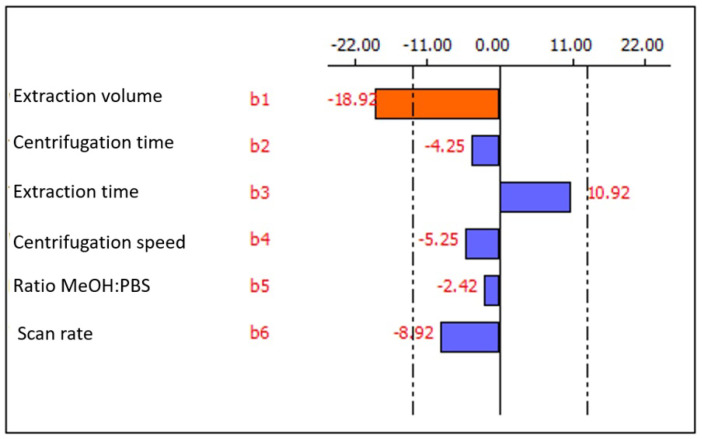
Pareto chart obtained in the Plackett-Burmann screening study.

When an analytical method is developed, it is necessary to optimize its experimental factors. The Plackett-Burmann design was used to construct the matrix of experiments, including six factors (Table S1) in twelve runs (Table S2). The Pareto chart of this screening study is shown in Figure 2. In this chart, bars that exceed dashed lines can be considered significant with 95% probability. In addition, rightward bars indicate a positive effect on the response when increasing the corresponding factor from a lower to a high level, while leftward bars indicate a negative effect on the response when passing from a lower to an upper level of the corresponding factor. 

From the Pareto chart it is possible to obtain information on the best conditions to perform the electrochemical determination. In the first place, the extraction time stands out as the only factor that presents a positive effect, however, its effect is not significant. The rest of the factors show negative effects, being the extraction volume the only significant factor. Therefore, it was necessary to perform a univariate study at different extraction volumes, keeping constant the non-significant factors at the most favorable level. 

To perform this study, different volumes were set around the previous lower level (50 mL). Extractions were performed with different volumes of MeOH, both with and without spiking, to obtain the recovery factors. The results obtained for the recovery factor during the univariate study are shown in Table S3. 

The Table S3 shows that the optimum volume is 40 mL of extractant, since a higher value is obtained in the recovery factor determined by the electrochemical method. Therefore, the optimum conditions for all the experimental factors are: extraction volume: 40 mL, extraction time: 10 min, centrifugation time: 2 min, centrifugation speed: 1000 rpm, ratio PBS:MeOH 1:1 and scan rate: 50 mV s^−1^. 

These are the conditions under which all determinations were performed with the two electrochemical sensors developed. 

### 3.3. Evaluation of Analytical Figures of Merit

To evaluate the analytical figures of merit by CV, a study was carried out by cyclic voltammetry at a scan rate of 50 mV s^−1^ between 0 and 1.8 V, using a screen-printed carbon electrode in the different MTU standard solutions. 

**Figure 3:**
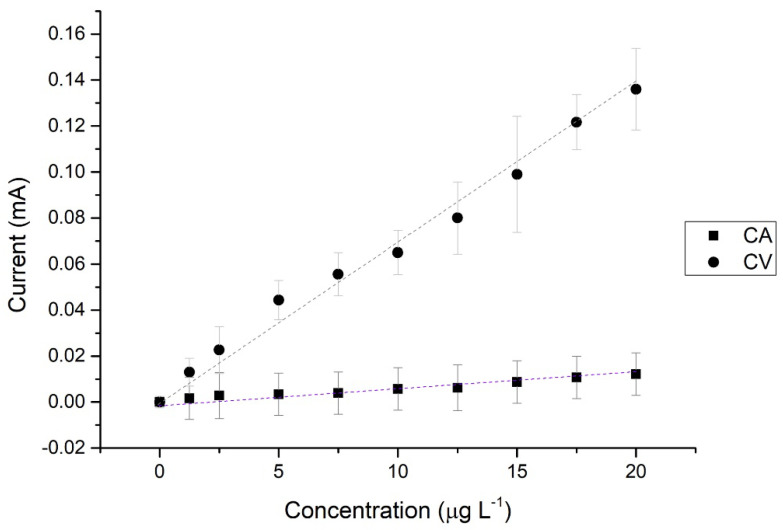
Calibration curves obtained by Cyclic Voltammetry (CV) and Chronoamperometry (CA). PBS:MeOH 1:1. Carbon SPE.

By plotting the current of each of the standards at a potential of 1.55 V as a function of concentration, it is possible to construct a calibration curve (Figure 3). 

Once the potential in which MTU oxidation occurs was determined, the next study was carried out by means of chronoamperometry (amperometric sensor), measuring how the current intensity changes with time at different concentrations. To compare with the voltammetric method, the same potential was chosen as in the previous section: 1.55 V. 

The value of the current, once a steady state chronoamperogram is obtained, can be plotted as a function of concentration (Figure 3). 

The electrodes used in the electrochemical sensors developed were commercial screen-printed electrodes with carbon ink that can be used once. In this work, commercial electrodes of different batches were used. Therefore, each of the electrodes could initially present different composition, which leads to large relative errors when performing replicates of the calibration curves. However, these relative errors do not significantly affect the results provided by the developed electrochemical sensors.

By plotting the absorbance of each of the standards at a wavelength of 435 nm as a function of concentration, it is possible to construct a calibration curve of the UV-Vis spectrophotometry method (Figure S3b).

For a better comparison, the figures of merit of the two developed electrochemical methods based on screen-printed carbon electrodes along with the method based on UV-Vis spectrophotometry are presented in Table 1. 

Comparing the linearities obtained (Table 1), it is observed that one of the electrochemical methods, chronoamperometry (CA), presents the worst coefficient of correlation value. This method also has the highest value of coefficient of variation. However, the comparison of the electrochemical methods with the UV-vis spectrophotometry one reveals that both electrochemical methods show higher sensitivity. Moreover, when the LOD and the LOQ of the three procedures are compared, the best values are obtained in the electrochemical ones with values two order of magnitude lower. 

As it is shown in Table 1, the coefficients of variation of the electrochemical methods are larger in percentage but if the concentrations of MTU are compared, the errors are lower than the obtained errors of UV-Vis spectrophotometric method. This means that the errors in the developed methods are acceptable for the miniaturized electrochemical sensors.

#### Comparison with Other Published Methods in Literature

A comparison of the results obtained in this work with other relevant methods used in the MTU determination previously reported in literature is summarized in Table 2. 

As can be seen in Table 2, most of the methods previously reported are based on chromatography associated with some detection technique. This characteristic is the main advantage over the sensors developed in this work, since with the amperometric and voltammetric sensors only MTU is determined, whereas the chromatographic methods allow the determination of multiple analytes in the same analysis. 

However, the LOD obtained in this work is lower than the previously reported by other authors, and it is obtained with a simple and cheap instrumentation. 

From the point of view of sample preparation, all the reported methodologies require extraction of the analyte from the matrix, since they are not able of determine MTU directly from the meat sample. It can be established as a common characteristic for all of them that extraction is carried out using different organic solvents such as acetonitrile or methanol and in relatively high quantities, approximately 50 mL. Most of the previously reported procedures require the combination of several high-hazard organic solvents to perform the extraction and purification prior to detection. However, only the HPLC-MS/MS [18] methodology and the one developed in this work avoid these steps. 

Moreover, considering that most chromatographic methodologies require derivatization prior to detection, the developed electrochemical sensors are the only method that allows the determination in aqueous medium without derivatization of the analyte. 

The above characteristics of the electrochemical determination, in addition to the low sample volume necessary to perform the detection, as well as the short time required and the relatively low cost of the electrochemical instrumentation, make it a good and promising methodology that could be implemented to improve and speed up the determination of MTU in meat samples.

### 3.4. Method Applicability

It was necessary to check the applicabiity of the MTU extraction procedure in meat samples for consumption, because the method has not been previously tested. Thus, to validate the procedure, the real meat samples should be spiked, and the recovery should be calculated [34].

The enrichment procedure consists of adding the analyte to the solid sample and then carrying out the extraction process. And after the extraction process, the recovery factor is calculated. Then, to know what type of calibration was necessary, the samples were spiked, and their concentration were determined by external calibration (Table 3) and using standard addition calibration (Table S4). The spiked concentration was 50 µg L^−1^ which, after the dilution, is in the linear working range of MTU concentrations (0 – 20 µg L^−1^). 

No significant differences are observed between the recovery values obtained by external calibration and by standard addition calibration, so it can be concluded that it is not necessary to perform the standard addition calibration since there were no serious matrix effects that falsify the results obtained. Because of this, the following determinations were performed using external calibration, which is an advantage since it reduces the analysis time.

### 3.5. Comparison with UV-Vis Spectrophotometric Determination

The electrochemical determination methods are compared with UV-Vis spectrophotometric determination method using the same real samples (Table S5). 

The results obtained are compared by means of a significance test (Fisher’s test) that measures the differences between the variances of two sets of values [35].

In this work the results obtained by the two electrochemical sensors are compared, as well as the values provided by each of the electrochemical techniques with the spectrophotometric method. 

Table S6 shows the values obtained after applying Fisher’s test. 

From the values obtained, several conclusions can be obtained: (i) No significant statistical differences are observed between the results obtained using the two electrochemical sensors under the conditions studied; (ii) No significant differences in the results obtained with the chronoamperometric sensor and the spectrophotometric method in the two types of matrixes analyzed. However, differences are observed when comparing the spectrophotometric method with the voltammetric sensor in the beef liver sample. The spectrophotometric method can determine several analytes of the thiouracil family, and the existence of significant statistical differences between the results could indicate the presence of another thiouracil compound in the sample. 

### 3.6. Application of the Analytical Eco-Scale

An ideally sustainable analysis should be characterized by the total, partial elimination or reduction of reagent use, minimization of energy consumption and no waste generation. Then, the sustainability of the new analytical method using electrochemical determination has been analyzed. 

Several methods are developed to evaluate quantitatively the sustainability of analytical methodologies, including the Analytical Eco-Scale [36].

In this Eco-Scale, the more sustainable and environmentally friendly the process, the higher the final score obtained. By establishing these criteria, the analytical processes can be classified as excellent (>75), acceptable (50–75) and inadequate (<50). 

Table 4 shows the total score obtained for the different procedures used in this work according to the analytical Eco-Scale. The breakdown of penalty points (PP) is attached in the Supplementary Material of this article. 

As shown in Table 4, the score obtained for the UV-Vis spectrophotometry method (official method) is less than 50, so the methodology can be categorized as inadequate from the point of view of sustainability. In contrast, the electrochemical methods are in the excellent category with a score higher than 75, although close to its limit. This indicates that the development of this methodology may be the beginning for the implementation of more sustainable determination processes, but further studies are required. For example, the optimization of the conditions of the electrochemical sensors in droplet is necessary to increase their degree of ecology.

## 4. Conclusions

The electrochemical techniques studied in this work, chronoamperometry and cyclic voltammetry, together with the use of commercial screen-printed carbon electrodes, are used in the determination of a veterinary drug such as MTU in real meat samples. The methodology used is adequate for MTU determination in real samples, recording similar results than those provided by UV-vis spectrophotometry determination. Comparing the methodology developed with previously published procedures in literature, it is concluded that it is the one with the best detection limit. Moreover, the electrochemical methodology is the only one that allows determination in aqueous medium without the need for any derivatization which is an advantage in comparison with UV-vis spectrophotometric determination within others. Moreover, the electrochemical determination requires portable instrumentation with lower cost in comparison to methods used in the literature. However, its main disadvantage is the impossibility of performing multiple analyte determinations on the same sample, unlike techniques that use chromatography. 

The great advantage of using SPE and electrochemical determination is the low sample volume required for the analysis (100 µL). However, it is necessary to change the solvent at the sample preparation stage. Then, this change of medium, from organic to aqueous solvent, can lead to an increase in experimental errors, although it makes the method more sustainable. 

After the optimization of the most significant variables by means of a multivariate method both in the extraction of the samples and in the electrochemical detection, the optimal conditions for the methodology are obtained, managing to reduce the volume of MeOH, used as extractant, and the preparation time. 

The MTU concentrations determined by electrochemical methods in meat samples are lower than the legal limit established by legislation and the results agree with the UV-Vis spectrophotometric method. 

The results obtained for electrochemical determination do not show significant statistical differences for the two electrochemical techniques investigated, and therefore one or the other can be selected indistinctly for analysis of MTU in real samples. 

After the application of the analytical Eco-Scale, it can be assured that the method developed by means of electrochemical sensors using commercial screen-printed carbon electrodes is more sustainable and eco-friendlier than the method UV-Vis spectrophotometry method considered as official. 

The future challenge before the implementation of these electrochemical sensors is the decrease of the volume of extractant, since the volume needed to perform the determination is small, around 100μL. The use of less harmful solvents, which generate less non-degradable residues and less toxicity, such as ionic liquids or DES (Deep Eutectic Solvents), should also be studied. All these are now under investigation. 

## Figures and Tables

**Table 1:** Analytical figures of merit of the methods for MTU determination.

Parameters	CV	CA	UV-Vis Spectrophotometry
r	0.9957	0.9823	0.9956
Sensitivity (μA L µg^−1^)	6.49 ± 0.03	2.273 ± 0.015	0.36 ± 0.02 ^a^
LOD (µg L^−1^) ^b^	0.13	0.13	15
LOQ (µg L^−1^) ^b^	0.4	0.4	50
C.V. (%) ^c^	16	30	6

^a^ In a.u. L µg^−1^. ^b^ The LOD and LOQ is empirically determined [33]. ^c^ Three replicates have been performed for each of the experiments. [MTU]_CV/CA_ = 0.13 µg L^−1^ and [MTU]_UV-Vis_ = 15 µg L^−1^.

**Table 2:** Comparative performance of some methods used in the MTU determination in meat samples reported in literature.

Analytes	Samples	Method	Derivatization	LOD (μg L^−1^)	References
TU, MTU, ETU, DMTU, PTU, PhTU, BTU, MBI	Biological matrices, including bovine and porcine liver	LC-ESI-MS/MS	Yes, 3-iodobenzyl bromide	1.6	[17]
MBI, TU, MTU, PTU, PhTU	Beef meat	GC-MS-SIM	Yes, PFBBr under basic conditions	2	[16]
TU, MTU	Pork meat	LC-ED	No	10	[15]
TU, MTU, PTU, PhTU, TAP	Bovine meat	CE	Yes, under basic conditions	20	[18]
TAP, TU, MTU, PTU, PhTU	Beef meat	LC-MS/MS (QqQ)	Yes, 3-iodobenzyl bromide	0.80	[14]
MTU, PTU	Bovine meat	GC-FPD	Yes, methylation reaction	10	[13]
TAP, TU, MTU, PTU, PhTU	Beef meat	LC-MS/MS (IT)	Yes, 3-iodobenzyl bromide	2.07	[14]
MBI, PTU, PhTU, TAP, TU, MTU	Liver (pork, beef, venison, rabbit)	HPLC-MS/MS	No	5	[18]
MTU	Beef liver and foie	CV and CA	No, only change of medium	0.13	This work

TU: 2-thiouracil, MTU: 6-methyl-2-thiouracil, ETU: 6-ethyl-2-thiouracil, DMTU: 5,6-dimethyl-2-thiouracil, PTU: 6-propyl-2-thiouracil, PhTU: 6-phenyl-2-thiouracil, BTU: 6-benzyl-2-thiouracil, MBI: 2-mercaptobenzimidazole, TAP: 1-methyl-2-mercaptoimidazole, PFBBr: pentafluorobenzyl bromide, LC-ESI-MS/MS: Liquid chromatography with electrospray ionization coupled to tandem mass spectrometry, GC-MS-SIM: Gas chromatography coupled to ion selective mode mass spectrometry, LC-ED: Liquid chromatography with electrochemical detection, CE: Capillary electrophoresis, LC-MS/MS (QqQ):Liquid chromatography coupled tandem mass spectrometry with triple quadrupole, GC-FPD: Gas chromatography with flame photometric detector, LC-MS/MS (IT): Liquid chromatography coupled ion trap tandem mass spectrometry, HPLC-MS/MS: High performance liquid chromatography coupled tandem mass spectrometry, CV: Cyclic Voltammetry, CA: Chronoamperometry.

**Table 3:** Analytical results obtained in the analysis of two spiked samples by the analytical method proposed using external calibration. The concentration and recovery values are expressed as the mean ± standard deviation of three replicates.

		Cyclic Voltammetry		Chronoamperometry	
Sample	Spiked Value (µg L^−1^)	Found (µg L^−1^)	R (%)	Found (µg L^−1^)	R (%)
Beef liver	-	66 ± 6	-	67 ± 18	-
	50	113 ± 8	94 ± 3	110 ± 21	85 ± 6
Foie	-	73 ± 17	-	70 ± 25	-
	50	121 ± 18	95 ± 2	114 ± 29	88 ± 8

**Table 4:** Total score and penalty points (PP) of the analytical methods evaluated after the application of the Analytical Eco-Scale.

UV-Vis Method		Electrochemical Sensors	
	PP		PP
Reagents	40	Reagents	14
Waste	8	Waste	8
Energy consumption	3	Energy consumption	0
Occupational hazard	3	Occupational hazard	1
Score	46	Score	77

## Data Availability

Not applicable.

## Supplementary Materials

The following supporting information can be downloaded at: https://www.mdpi.com/article/10.3390/s22228842/s1, Figure S1: Scheme of the extraction procedure; Figure S2: Scheme of the determination procedure by UV-Vis spectroscopy; Figure S3a: UV-Vis spectra of solutions that contains different concentrations of MTU in CHCl3 (16.3, 49.3, 82.3 and 164.5 μg L−1); Figure S3b: Calibration curve of MTU in CHCl3 obtained by UV-Vis spectrophotometry (λ = 435 nm); Figure S4: Cyclic voltammograms during the 5th cycle of carbon SPE in a solution that contains different concentrations of MTU (0, 2.5, 7.5 and 17.5 μg L−1). MeOH:PBS (50:50) solution, v = 50 mV s−1; Figure S5: Chronoamperometry at 1.55 V for a carbon SPE in a solution that contains different concentrations of MTU (0, 2.5, 7.5 and 17.5 μg L−1). MeOH:PBS (50:50) solution; Figure S6: Cyclic voltammogram during the 5th cycle of carbon SPE in a solution that contains the extract of beef liver sample. MeOH:PBS (50:50) solution, v = 50 mV s−1; Table S1: Factors and levels of Plackett-Burmann design; Table S2: Matrix of experiments in the Plackett-Burmann design; Table S3: Effect of the extractant volume in MTU determination; Table S4: Analytical results obtained in the analysis of two spiked samples by the analytical method proposed using standard addition; Table S5: Analytical results obtained by the three analytical methods used in this work; Table S6: Application of Fisher’s test to determine the existence of statical differences of the results obtained from the different methods used; Table S7: Assignment of penalty points (PP) for the UV-Vis spectrophotometric method; Table S8: Assignment of penalty points (PP) for the miniaturized electrochemical sensors-based method.
