# Supplementary material for: Electrochemical Sensor for the Determination of Methylthiouracil in Meat Samples
Source: Sensors (Basel). 2022 Nov 15;22(22):8842. doi: 10.3390/s22228842 (PMC9696097; doi:10.3390/s22228842)
Supplement: Supplementary file 1 [file sensors-22-08842-s001.zip › sensors-1979546-supplementary.pdf]

Article

Electrochemical Sensor for the Determination of Methylthiouracil in Meat Samples

Andrea Marco [1], Antonio Canals [2,*], Emilia Morallón [1] and Miguel Ángel Aguirre [2]

[1] Departamento de Química Física e Instituto Universitario de Materiales, Universidad de Alicante, Apartado 99, Alicante 03080, Spain
[2] Departamento de Química Analítica, Nutrición y Bromatología e Instituto Universitario de Materiales, Universidad de Alicante, Apartado 99, Alicante 03080, Spain
* Correspondence: a.canals@ua.es

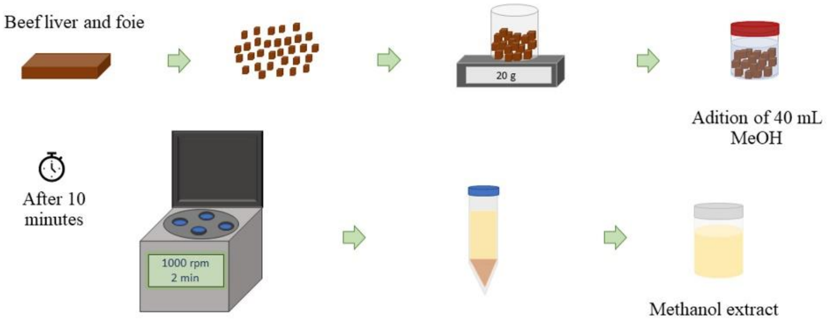

Figure S1. Scheme of the extraction procedure.

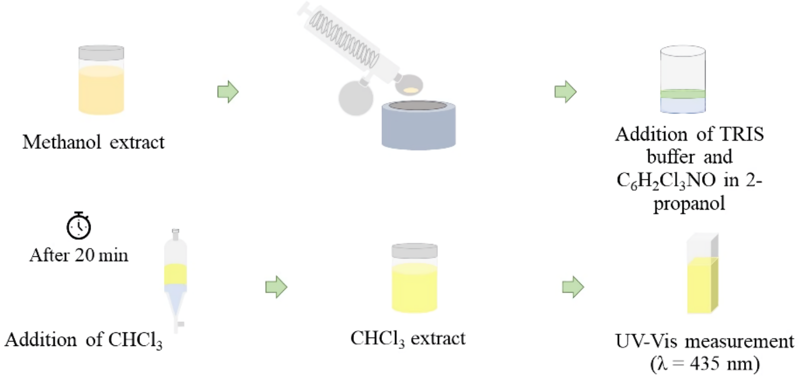

Figure S2. Scheme of the determination procedure by UV-Vis spectroscopy.

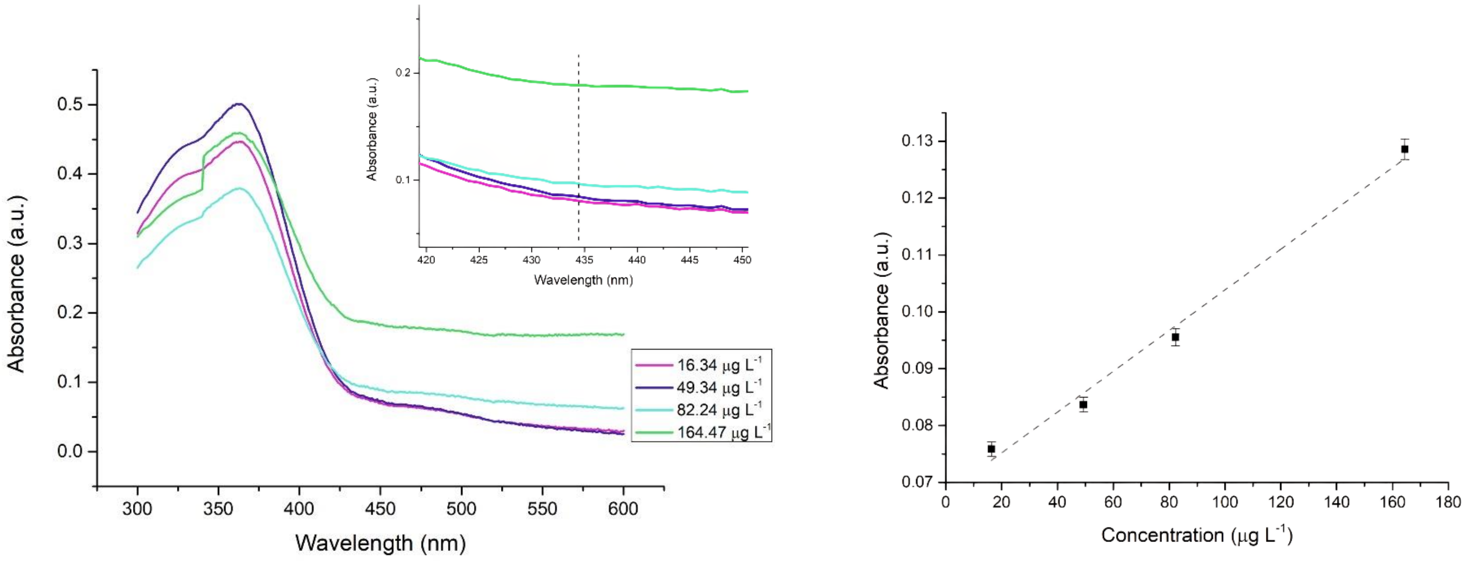

(**a**)

(**b**)

Figure S3. (**a**) UV-Vis spectra of solutions that contains different concentrations of MTU in $CHCl_3$ (16.3, 49.3, 82.3 and 164.5 μg L^{-1}). (**b**) Calibration curve of MTU in $CHCl_3$ obtained by UV-Vis spectrophotometry (λ= 435 nm).

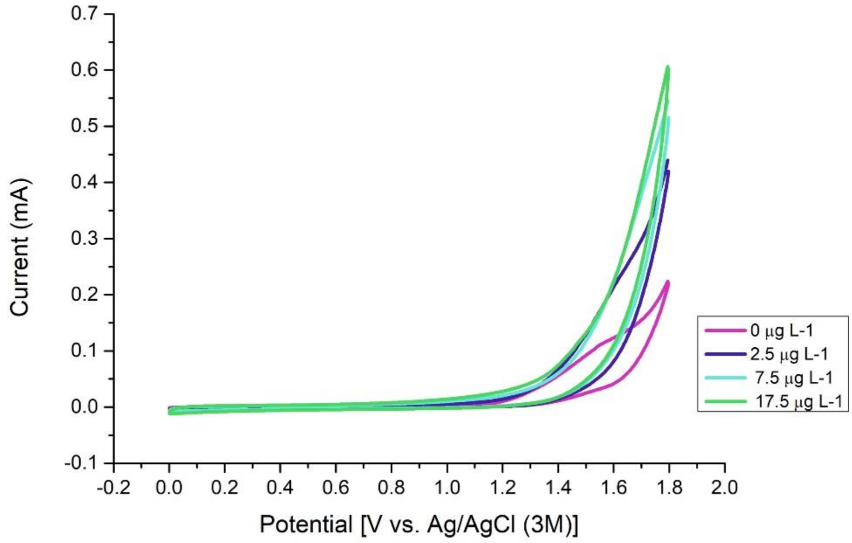

Figure S4. Cyclic voltammograms during the 5th cycle of carbon SPE in a solution that contains different concentrations of MTU (0, 2.5, 7.5 and 17.5 μg L^{-1}). MeOH:PBS (50:50) solution, v = 50 mV s^{-1}.

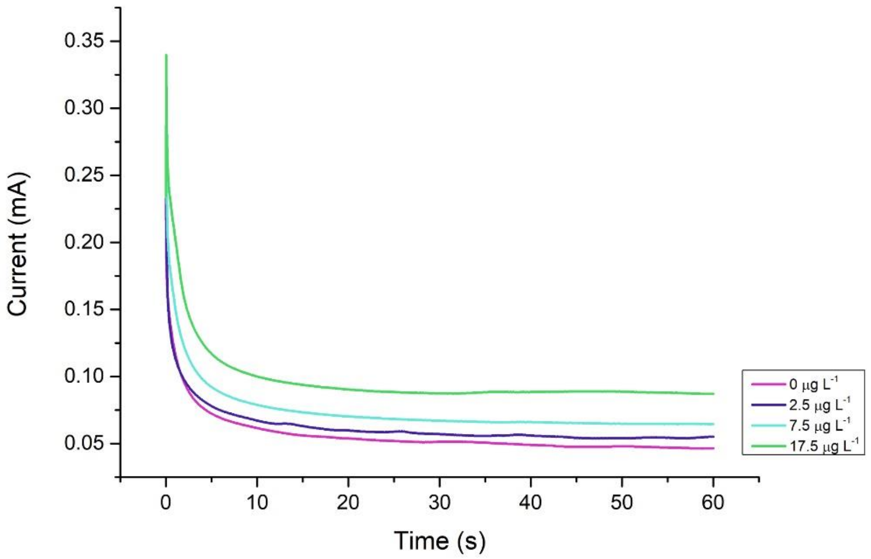

Figure S5. Chronoamperometry at 1.55 V for a carbon SPE in a solution that contains different concentrations of MTU (0, 2.5, 7.5 and 17.5 μg L^{-1}). MeOH:PBS (50:50) solution.

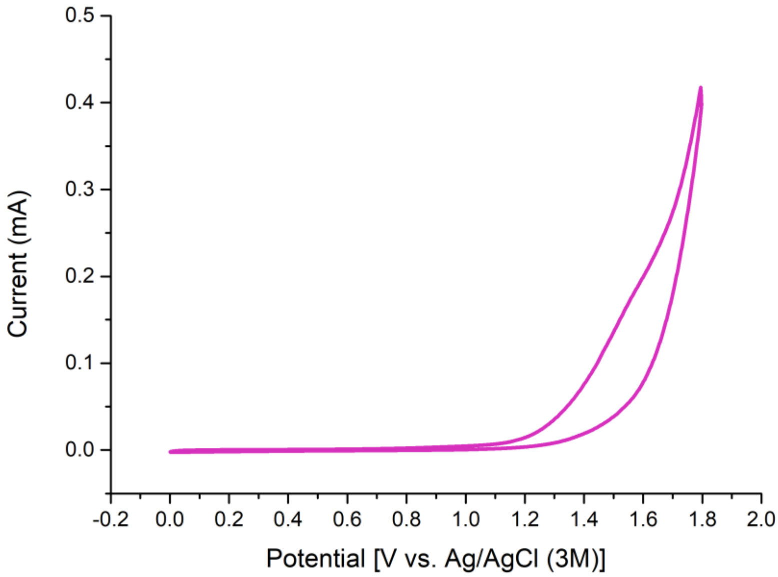

Figure S6. Cyclic voltammogram during the 5th cycle of carbon SPE in a solution that contains the extract of beef liver sample. MeOH:PBS (50:50) solution, v = 50 mV s^{-1}.

Table S1. Factors and levels of Plackett-Burmann design.

Factor	Level	
	Lower (-1)	Upper (+1)
Extraction volume (mL)	50	100
Centrifugation time (min)	2	5
Extraction time (min)	5	10
Centrifugation speed (rpm)	1000	2000
Ratio MeOH:PBS	1:1	1:4
Scan rate (mV s^{-1})	50	75

Table S2. Matrix of experiments in the Plackett-Burmann design.

Experiment	Extraction volume (mL)	Extraction time (min)	Centrifugation time (min)	Centrifugation speed (rpm)	Ratio MeOH:PBS	Scan rate (mV s^{-1})
1	100	10	2	2000	1:4	75
2	50	10	5	1000	1:4	75
3	100	5	5	2000	1:1	75
4	50	10	2	2000	1:4	50
5	50	5	5	1000	1:4	75
6	50	5	2	2000	1:1	75
7	100	5	2	1000	1:4	50
8	100	10	2	1000	1:1	75
9	100	10	5	1000	1:1	50
10	50	10	5	2000	1:1	50
11	100	5	5	2000	1:4	50
12	50	5	2	1000	1:1	50

Table S3. Effect of the extractant volume in MTU determination.

Extraction volume (mL)	Recovery factor (%) *
30	75
40	98
50	95
60	90
70	83

*The experimental conditions for the analytical procedure are: extraction time: 10 minutes, centrifugation time: 2 minutes, centrifugation speed: 1000 rpm, ratio PBS:MeOH: 1:1, scan rate: 50 mV s^{-1}.

Table S4. Analytical results obtained in the analysis of two spiked samples by the analytical method proposed using standard addition. The concentration and recovery values are expressed as the mean ± standard deviation of three replicates.

Sample	Spiked value (µg L^{-1})	Cyclic Voltammetry		Chronoamperometry	
		**Found (µg L^{-1})**	**R (%)**	**Found (µg L^{-1})**	**R (%)**
Beef liver	-	72 ± 8	-	69 ± 9	-
	50	119 ± 10	93 ± 5	114 ± 13	89 ± 8
Foie	-	70 ± 11	-	75 ± 19	-
	50	118 ± 13	95 ± 4	123 ± 21	96 ± 4

Table S5. Analytical results obtained by the three analytical methods used in this work. Found concentration values are expressed as the mean ± standard deviation of three replicates.

	**Cyclic Voltammetry**	**Chronoamperometry**	**UV-Vis Spectrophotometry**
**Sample**	**Found (µg L^{-1})**	**Found (µg L^{-1})**	**Found (µg L^{-1})**
Beef liver	66 ± 6	67 ± 18	92 ± 4
Foie	73 ± 17	70 ± 25	96 ± 7

Table S6. Application of Fisher's test to determine the existence of statical differences of the results obtained from the different methods used.

**Cyclic Voltammetry vs Chronoamperometry**					
**Sample**	**F_{calc}**	**F_{tab}**	**t_{calc}**	**t_{tab}**	Null Hypothesis
Beef liver	10.28	39	0.07	4.303	**True**
Foie	2.24	39	0.16	4.303	**True**
Cyclic Voltammetry vs UV-Vis Spectrophotometry					
Sample	F_{calc}	F_{tab}	t_{calc}	t_{tab}	Null Hypothesis
Beef liver	1.95	39	7.22	4.303	**False**
Foie	6.74	39	2.99	4.303	**True**
Chronoamperometry vs UV-Vis Spectrophotometry					
Sample	F_{calc}	F_{tab}	t_{calc}	t_{tab}	Null Hypothesis
Beef liver	20.03	39	1.89	4.303	**True**
Foie	15.07	39	1.42	4.303	**True**

S1. Application of Analytical Eco-Scale

For the application of the analytical Eco-Scale to the different methodologies used for the determination of the MTU, the worst possible conditions, that is, the least sustainable ones, are considered.

For the assignment of penalty points (PP) due to the use of reagents, penalties are assigned for both the hazard and the quantity, being the final penalty in this section the product of the penalties in both categories. The criteria followed for assigning points in the hazard category is to take into account the number of pictograms assigned to each of the reagents.

According to the ecological scale, an ideal analysis procedure has an initial score of 100. However, from this score, it is necessary to subtract the penalty points assigned to the variables of the method that differ from the sustainable ideality. Therefore, the total score initially assigned to the methodology is reduced due to non-ecological variables. The PP are assigned for the reagents and their hazard (physical, environmental, and occupational), as well as for the amount of energy used and the amount of waste generated in the total analytical process.

First, the degree of ecology of the UV-Vis spectrophotometric method used for the determination of thiouracils is evaluated (Table S7). In this methodology, various organic reagents of different levels are used with different hazards and in relatively large quantities. In addition, toxic vapours are produced, which increases the occupational risk, and it has a higher energy consumption, due to the need to use the rotary evaporator and the constant use of extraction hoods.

Table S7. Assignment of penalty points (PP) for the UV-Vis spectrophotometric method.

**Reagents**	
	PP
MeOH: 100 mL + 250 mL	3 x 6
Chloroform: 10 mL	2 x 4
Dichloroquinone-chloroimide: 20 mg	1 x 4
MTU: 25 mg	1 x 2
TRIS buffer: 20 mL	2 x 0
2-propanol: 50 mL	2 x 4
Instruments	
	PP
Extraction hood (3677 W, 1 h)	1
Centrifugue (550 W, 5 min)	0
Rotary evaporator (1300 W, 15 min)	1
UV-Vis spectrophotometer (130,5 W, 1h)	1
Occupacional hazards	3
Waste	8

The sustainability of the electrochemical sensor method developed with SPE is then quantified to demonstrate its higher degree of ecology compared to other determination methods (Table S8). In this procedure, the determination is carried out in aqueous media, thus reducing occupational hazards, and using instruments with low energy consumption.

Table S8. Assignment of penalty points (PP) for the miniaturized electrochemical sensors-based method.

**Reagents**	
	PP
MeOH: 40 mL + 50 mL	2 x 6
MTU: 0,300 mg	1 x 2
Potassium phosphate: 6,8 g	1 x 0
Tryhidrate dipotassium phosphate: 11,4 g	2 x 0
Instruments	
	PP
Extraction hood (3677 W, 15 min)	0
Centrífugue (550 W, 2 min)	0
Potenciostat (40 W, 2 h)	0
Occupacional hazards	1
Waste	8
